# Effectiveness of sensor monitoring in an occupational therapy rehabilitation program for older individuals after hip fracture, the SO-HIP trial: study protocol of a three-arm stepped wedge cluster randomized trial

**DOI:** 10.1186/s12913-016-1934-0

**Published:** 2017-01-03

**Authors:** Margriet C. Pol, Gerben ter Riet, Margo van Hartingsveldt, Ben Kröse, Sophia E. de Rooij, Bianca M. Buurman

**Affiliations:** 1 Research Group Occupational Therapy, ACHIEVE, Centre of Applied Research, Faculty of Health, Amsterdam University of Applied Sciences, Room B 122, Tafelbergweg 51, PO Box 2557, 1000CN Amsterdam, The Netherlands; 2 Department General Practice, Academic Medical Center, University of Amsterdam, Amsterdam, The Netherlands; 3 Research Group Digital Life, Amsterdam University of Applied Sciences, University of Amsterdam, Amsterdam, The Netherlands; 4 Department of Internal Medicine, Geriatric Section, Academic Medical Center, University of Amsterdam and The University Medical Center Groningen (UMCG), Amsterdam, The Netherlands; 5 Department of Internal Medicine, Section of Geriatric Medicine, Academic Medical Center, University of Amsterdam, Amsterdam, The Netherlands

**Keywords:** Sensor monitoring, Hip fracture, Occupational therapy, Coaching, Effectiveness, Stepped wedge randomized trial, Activities of daily living

## Abstract

**Background:**

The performance of activities of daily living (ADL) at home is important for the recovery of older individuals after hip fracture. However, 20–90% of these individuals lose ADL function and never fully recover. It is currently unknown to what extent occupational therapy (OT) with coaching based on cognitive behavioral treatment (CBT) improves recovery. The same holds for sensor monitoring-based coaching in addition to OT. Here, we describe the design of a study investigating the effect of sensor monitoring embedded in an OT rehabilitation program on the recovery of ADL among older individuals after hip fracture.

**Methods/ Design:**

Six nursing homes will be randomized in a three-arm stepped wedge cluster randomized trial. All nursing homes will initially provide standard care. At designated time points, nursing homes, successively and in random order, will cross over to the provision of OT and at the next time point, to sensor monitoring-enhanced OT. A total of 288 older individuals, previously living alone in the community, who after a hip fracture were admitted to a geriatric rehabilitation ward for a short-term rehabilitation, will be enrolled.

Individuals in the first intervention group (OTc) will participate in an OT rehabilitation program with coaching based on cognitive behavioral therapy (CBT) principles. In the sensor monitoring group, sensor monitoring is added to the OT intervention (OTcsm). Participants will receive a sensor monitoring system consisting of (i) an activity monitor during nursing home stay, (ii) a sensor monitoring system at home and a (iii) a web-based feedback application. These components will be embedded in the OT. The OT consists of a weekly session with an occupational therapist during the nursing home stay followed by four home visits and four telephone consultations. The primary outcome is patient-perceived daily functioning at 6 months, assessed using the Canadian Occupational Performance Measure (COPM).

**Discussion:**

As far as we know, this study is the first large-scale stepped wedge trial, studying the effect of sensor monitoring embedded in an OT coaching program. The study will provide new knowledge on the combined intervention of sensor monitoring and coaching in OT as a part of a rehabilitation program to enable older individuals to perform everyday activities and to remain living independently after hip fracture.

**Trial registration number:**

Netherlands National Trial Register, NTR 5716

Date registered: April 1 2016

## Background

Each year in the Netherlands, 17,000 people are admitted to a hospital after a hip fracture. The effects of a hip fracture are serious; one year after a hip fracture, 25% of patients have died and 20–90% of older individuals have new Activities of Daily Living (ADL) disabilities, defined as a functional decline [1–3]. Risk factors for functional decline after hip fracture can be divided into non-modifiable and modifiable risk factors. Non-modifiable risk factors are older age, female gender, living alone, cognitive impairment (dementia) and comorbidities. The modifiable risk factors are activities of daily living (ADL), walking ability, and depression [4–6]. Psychological factors such as low levels of self-efficacy and fear of falling have also been associated with functional decline after hip fracture in older individuals [7, 8].

Currently, most multidisciplinary rehabilitation programs for patients after hip fracture concentrate on improving mobility and ADL function but not fear of falling [7]. The evidence on the effectiveness of these rehabilitation programs on the recovery of ADL function is mixed. Exercise interventions have been used to improve physical function (e.g., gait speed, mobilization, balance, and strength), but despite an improvement in physical function, many older persons do not achieve a full recovery of ADL function [9, 10]. High-intensity (e.g., 4 times a week physical therapy) and intensive extended supervised exercise programs (e.g., during 12 month) had a significant impact on various physical functions, but the cost-effectiveness of these extended programs is unclear [11]. The main component of effective studies is ‘home-based functional task exercises’ (e.g., walking stairs, transferring), which results in a modest improvement in physical function post-discharge or at one year after discharge [12].

Fear of falling may have an important influence on functional recovery after hip fracture [7]. Because of the fear of falling, people feel insecure while moving and performing activities of daily living, and as a consequence, they do less and less. However, for good recovery, performing ADLs is essential [7, 8, 13, 14]. Therefore, for older individuals, mobility is an essential aspect of quality of life and crucial for the preservation of independence [15]. Fifty percent or more of patients with hip fracture suffer from a fear of falling, resulting in a reduction in physical activities [7]. Therefore, in order to be successful, rehabilitation programs may need to focus on increasing self-efficacy concerning falls and fear of falling. Additionally, programs should focus on setting realistic goals for increasing the performance of daily activities, change the environment to reduce the fall risk and promote physical activity to increase strength and balance [13].

To coach patients in modifying their patterns of thoughts (cognition) and activities (behavior) that contribute to the fear of falling, CBT principles can be used, consisting of five steps, which together have been proven effective [13, 16–18]: 1) to educate individuals about being physically active and to stimulate physical activity and exercise, 2) to ascertain the amount of movement and physical activity during the day and give feedback, 3) to set realistic goals for the performance of daily activities, 4) to plan these activities, and 5) to evaluate progress.

New healthcare technologies, such as sensor monitoring, can assist healthcare professionals in coaching more effectively without increasing their time expenditure. The sensors provide an objective continuous measurement of daily functioning and provide automatic feedback via a web-based application [19]. This can be combined with the coaching of the daily functions of the client [20, 21]. Older individuals who had a sensor system in their home during a long period of time appreciated having sensors at home and indicated that the technology supported their ability to live an independent life and contributed to their sense of safety [22–25]. However, as far as we know, sensor technologies have not yet been used in the rehabilitation of older patients after hip fracture.

The aim of the present study is to investigate the effect of sensor monitoring, embedded in a multidimensional OT rehabilitation program, on the recovery of physical ADL function among community-dwelling participants after hip fracture 6 months after the start of the rehabilitation in the nursing home compared to OT without sensor monitoring and to standard care.

## Methods

### Design and setting

The study is a three-phase, cross-sectional, complete design (data are collected from each cluster throughout the trial), stepped wedge, cluster randomized trial (SW-CRT). Clusters are nursing homes, which are the units of randomization. Table 1 shows the design matrix of the trial.

**Table 1:**
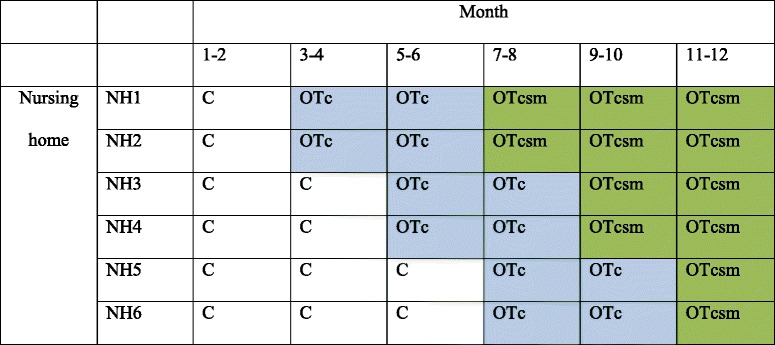
Design of the three-phase stepped wedge cluster randomized trial

*C* Care as usual, *OTc* Occupational therapy with coaching, *OTcsm* Occupational therapy with coaching and sensor monitoring, *NH* = *Cluster* = Nursing home

Trial duration =12 months (recruitment), 18 months (including exposure and measurements

Number of clusters = 6. Number of groups =3. Number of clusters per group =2 (cross over simultaneously)

Pre-rollout period = 2 months. Rollout period = 8 months. Post-rollout period = 2 months

Step length (intervention 1-2) = 2 months. Number of participants per step = 8

**Fig. 1:**
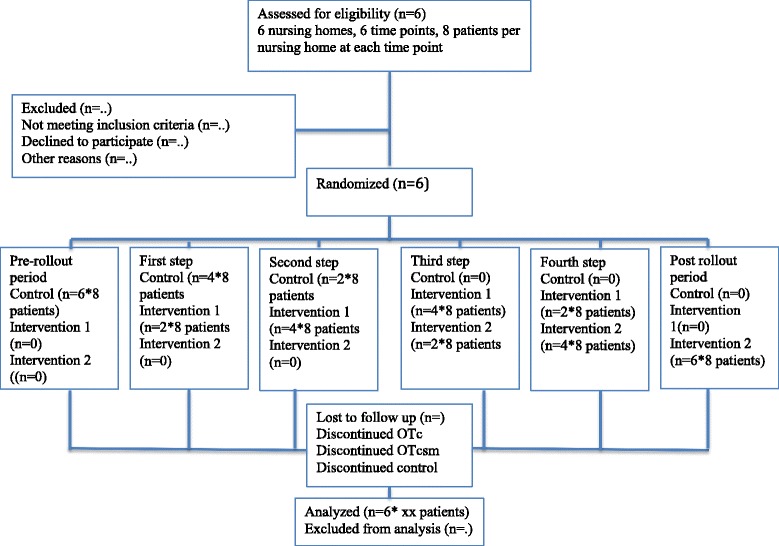
Flow of clusters and participants

Reporting following Copas et al 2015 (Trials, Fig. 1) [58]

Six clusters (nursing homes) will be randomized to one of three fixed sequences, each containing the three interventions. All clusters will start with providing standard care (control condition) at the beginning of the study. At predetermined time points, two clusters cross over from the control condition (C) to the first intervention, the OT intervention with coaching based on CBT (OTc). At other predetermined time points, two clusters crosses over to sensor monitoring embedded in an OT intervention based on CBT (OTcsm). The interval between the different time points will be 2 months. One advantage in terms of the willingness to participate applicability of the trial to the nursing homes is that all of the nursing homes will have implemented the intervention at the end of the study.

The feasibility study started October 20, 2015 with the Amaris Health group in two locations in Laren and Hilversum and will end September 2016. The methods and procedures are feasible. We made minor improvements to some of the procedures for the main study. The main study has started April 1, 2016 and will end September 2017. The following nursing homes, situated in the Northwest and Midwest part of the Netherlands, are involved in the main study: the Omring with locations in Hoorn and Lutjebroek, Magentazorg with locations in Alkmaar and Bergen, Amstelring with locations in Amstelveen and Hoofddorp, Zorgbalans with locations in Ijmuiden and Haarlem, Careyn with locations in Utrecht and Vinkeveen and Evean with a location in Zaandam and two locations in Amsterdam.

### Study population/eligibility

Nursing homes were invited to participate if they fulfill all of the following criteria: 1) have a geriatric rehabilitation ward for hip fracture rehabilitation, with a multidisciplinary team that consists of at least two OT professionals; 2) community-based occupational treatment is provided by the nursing home or can be provided by a community-based OT; and 3) are able to enroll at least 48 patients (8 patients per step) in total.

Participants are eligible if they meet the following criteria: 1) are admitted to a geriatric rehabilitation ward in a nursing home after hip surgery and have an indication for short term rehabilitation; 2) are at least 65 years old; 3) are living alone in the community or in a senior residence; 4) have a minimal-mental state examination (MMSE) score of 15 or higher (cognitive functioning).

Participants are excluded if at least one of the following applies: 1) terminal illness; 2) awaiting permanent placement in a nursing home; 3) no written informed consent.

### Recruitment of patients

After admission to the nursing home, the nursing home physicians will identify potential patients on the basis of the inclusion criteria. A research assistant will provide oral and written study information. The research assistant will contact interested patients and their caregiver(s) to provide further detailed information on the study and to check the inclusion criteria. Written informed consent obtained in the presence of the research assistant will be required prior to enrollment. A copy of the signed informed consent form will be given to the participant. The original signed consent document will be retained by the investigator. Then, baseline measurements will be performed.

All recruitment procedures will comply the Dutch Medical Research Involving Human Subjects Act and the WMA Declaration of Helsinki [26].

### Randomization procedure

Randomization was performed, 4 weeks before the start of the study, by the second author, who was not involved in the day to day logistics of care delivery. A dedicated program was written using the sample command in Stata version 13.1 (Stata Corp LP, College Station, TX) applying the following principles: (i) centers were ranked as to their size and likely patient recruitment potential; (ii) three strata were formed, 2 largest, 2 intermediate-sized and the 2 smallest centers; (iii) these were allocated in a way that would enhance the likelihood of collecting similar amounts of information the 3 strata across the 6 time periods; (iv) in particular, we forced the intermediate-sized centers in the 2-2-2 months periods; (v) we randomized the remaining 4 centers such that 1 large and 1 small center followed the 1-2-3 months periods and the other pair the 3-2-1 months periods, respectively.

Figure 1 shows the flow of clusters and participants through the trial using an adapted CONSORT diagram [27].

### The intervention

Table 2 shows the components of the standard care group (C) and the two intervention groups – OTc and OTcsm.

**Table 2:** Components of the control arm care as usual, OT with coaching and OT with coaching and sensor monitoring

	Time frame	Intervention component	Professional involved	Control arm	OTc	OTcsm
Nursing home	<48 h after admission	Geriatric assessment Preliminary care and treatment plan	Elderly care physician/Nurse	X	X	X
	Week 1	Multidisciplinary assessments	Nurse, PT, OT	X	X	X
	Week 2	Multidisciplinary care and treatment plan	Multidisciplinary team	X	X	X
	During NH	Multidisciplinary rehabilitation	Multidisciplinary team	X	X	X
	During NH	Wearing of the activity sensor	OT			X
	During NH	Once a week coaching by the sensor data	OT			X
	During NH	Once a week coaching	OT		X	
Home	<1 day after NH discharge	Installing sensor system and wearing activity monitor	Sensor installer			X
	Week 1	H1 Coaching	OT		X	X
	Week 2	H2 Coaching	OT		X	X
	Week 3	H3 Coaching	OT		X	X
	Week 4	H4 Coaching	OT		X	X
	Week 5, 6, Week 8, 10	Telephone consult Telephone consult	OT OT		X X	X X
	Week 12	Removal of the sensor system	Sensor installer			X

*OTc* Occupational therapy with coaching, *OTcsm* Occupational therapy with coaching and sensor monitoring, *NH* Nursing home, *PT* Physical therapist, *OT* Occupational therapist, *H1* Home visit 1, *H2* Home visit 2, *H3* Home visit 3, *H4* Home visit 4

#### Care as usual (C): rehabilitation provided to all patients included in the study

After admission to the nursing home, a multidisciplinary assessment including a consultation of the different disciplines begins. The multidisciplinary team in the nursing homes will comprise a nursing home physician, a nurse, a physical therapist (PT) and an occupational therapist (OT). If required, other professionals, such as a dietician or psychologist, will be consulted. Within 48 h after admission to the nursing home, the nursing home physician, together with the nurse, will conduct a comprehensive geriatric assessment and also coordinates wound care, pain management and the mobilization plan. S/he will also coordinate the patient’s multidisciplinary care and treatment team. The PT assessment will focus on mobility, muscle strength, balance transfer and walking. The OT assessment will focus on the performance of daily functions and safety at home. After the assessments, a multidisciplinary care and treatment plan will be made together with the patient. All patients will follow the evidence-based multidisciplinary rehabilitation program. Currently, in the Netherlands, the focus of rehabilitation after hip fracture is PT. Patients will be discharged after 3–6 weeks, as soon as they are able to function independently or with the assistance of formal or informal care at home. If needed, some of the patients receive rehabilitation at home or at a rehabilitation ward outside of the nursing home, but this is provided to a minority of patients.

#### Intervention arm 1: OT with coaching without sensor monitoring (OTc)

On top of the multidisciplinary rehabilitation, participants in this intervention group will receive an OT intervention with coaching (OTc). The primary role of OT is to optimize performance and engagement in meaningful activities and to improve participation. The OT interventions will focus on individual patients’ needs and include teaching patients strategies to improve task performance [28–31].

The coaching is based on evidence-based principles of a cognitive behavioral therapy (CBT) program concerning fear of falling [13, 22]. As fear of falling is very common in patients after hip fracture, a main aim is to reduce that fear and improve recovery. To coach patients in modifying their patterns of thought (cognition) and activities (behavior) that contribute to the fear of falling, the occupational therapist integrates the following five CBT principles (which have proven to improve fear of falling) in the rehabilitation: 1) to give information and education about the importance of physical activity and daily exercise; 2) to ascertain the amount of movement and physical activity during the day and give feedback 3) to define, together with the patient, realistic goals for the performance of daily activities; 4) to make an activity plan together with the patient and, if needed, practice exercises and daily activities in a safe manner accompanied by the occupational therapist. Patients will select the activities in which fear of falls are experienced that they consider relevant and important to practice; 5) to evaluate progress.

OT will take place once a week while a patient is still in nursing home. After discharge, the participants receive four home visits by an occupational therapist in the first 4 weeks after discharge, followed by four telephone consultations.

The first home visit takes place within 2 days after discharge from the nursing home and will cover changing to the environment to reduce fall risk and setting realistic goals for increasing daily physical activities. The duration of this first home-visit will be approximately 60 min.

The next, 45–60 min home visits in weeks 2, 3 and 4 will address the same five steps.

After the last visits in weeks 5, 6, 8, and 10 a 15-min telephone consultation is planned along the same lines.

#### Intervention arm II: OT with CBT-coaching using sensor monitoring as a coaching tool (OTcsm)

Participants in intervention arm II receive an OT intervention in which sensor monitoring is used to enhance coaching. The occupational therapist will use sensor monitoring as a tool to coach the patient during rehabilitation in the nursing home and as a ‘transitional care program’, focusing on the transition from the nursing home to the home during the post-discharge period.

#### Technical details of sensor monitoring using the SO-HIP tool

The SO-HIP tool consists of two different sensor systems: 1) a wearable activity monitor, and 2) a sensor monitoring system placed in the home of the participant (environmental sensor system). The development of the SO-HIP tool is based on the experiences in a preceding proof-of-concept by the University of Amsterdam and Amsterdam University of Applied Science (AUAS) that was started in 2011 [23, 32, 33].

**Fig. 2:**
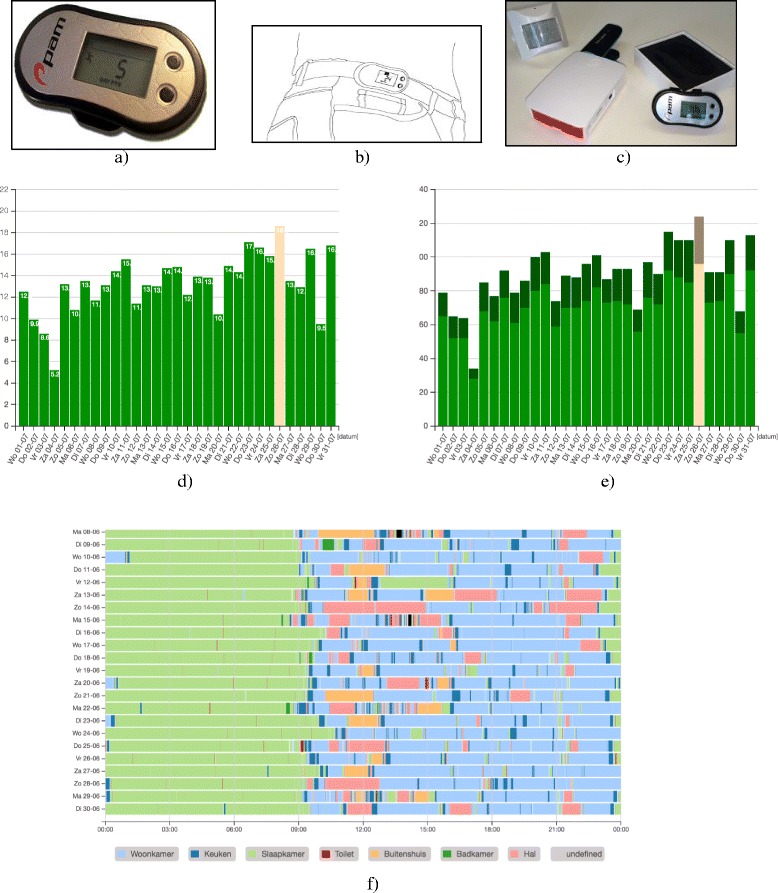
Sensor monitoring system and web application. **a** Pam sensor. **b** Pam sensor worn on a waist band. **c** Motion sensor, data box, pam sensor. **d** Measured movements per day expressed in a pam score. **e** The number of minutes active movements per day. **f** Visualization of an activity pattern measured by the wireless sensor monitoring system during one month. The different colors correspond with the different locations where activities took place. Each line corresponds with one day

1) The wearable activity monitor (Pam) (http://www.pamcoach.com) consists of a 3-dimensional accelerometer, 68 x 33 x 10 mm, wirelessly connected to a base unit from which the data are sent to a secure database and a web-based application (see Fig. 2). The Pam is worn on the hip and measures the time of all daily activities in minutes per day. We tested the feasibility. Older individuals experienced the pam is extremely easy to use: e.g. easy to clip on a waistband, comfortable to wear during the day and individuals don’t have to adjust anything to the device. The Pam measures the acceleration of the body movements and expresses the measured movements in the pam score. The Pam score is an index representing the ratio of energy expended through physical activity to resting metabolism [34].

The occupational therapist monitors these activities via a secure website and uses the sensor data as feedback for coaching the participant by following the five steps of CBT once a week during one of the rehabilitation sessions. In each visit, the progress with regard to physical daily activities will be discussed. A new goal will be set, and a discussion what happened during the week will take place, addressing what was easy, what were difficult activities, and why. (See further coaching details regarding the use of sensor monitoring).

2) The sensor monitoring system consists of a wireless sensor network with a base unit with 16 simple Benext sensors, covering the main spaces in a house. This system will be placed in the home when the participant is discharged from the nursing home. The sensors are passive infrared motion sensors (to detect motion in a specific area), contact switches (reed) on doors and cabinets (to measure whether doors are opened or closed), energy switch sensors (to measure the use of appliances such as the TV or washing machine), and one float sensor in the toilet (to measure the flushing of the toilet). The activity patterns of the daily functioning of participants are monitored using the wireless sensor monitoring system and are sent to a local base unit and stored in a secured database. These sensor data are analyzed by a computer program, which looks for activities of daily functioning and daily patterns in the data. (e.g., toileting or bathing can be recognized, but more complex activities such as preparing a breakfast, and other kitchen activities will also be recognized by the sensor system). A sequence of binary sensor data indicates the activity with the help of a recognition algorithm. The occupational therapist can use the reports of the sensor data via a secure web application to evaluate the daily functioning of the patient and by doing so appropriately coach the patient in performing daily functions and exercises following the same five steps learned during the nursing home rehabilitation (see Fig. 2).

The participants in the intervention OTcsm group receive information about the sensor monitoring at the start of the rehabilitation in the nursing home. This information includes a short manual and daily instruction on how to wear the activity monitor. In the week of discharge, the patients receive further information pertaining to the sensor monitoring at home.

#### Details for the use of sensor monitoring embedded in the OT intervention with coaching

From the start of the rehabilitation in the nursing home, the patient will wear an activity monitor (see technical details of the SO-HIP tool). The occupational therapist monitors the activities via a secure website and uses the sensor data as feedback for coaching the participant by following the five steps once a week during a coaching session. The sensor data reports can be used in the coaching as objective information about the current state of the amount of movement and activities performed during the day. The sensor data reports form a starting point for discussion about the daily patterns and activities that are important to practice and for making new realistic plans for activities based on the objective reports. The daily and weekly reports of the sensor data can also be used to evaluate progress of the rehabilitation.

During the rehabilitation in the nursing home, the patient learns, with the help of the occupational therapist, to make use of the sensor monitoring by following the five consequent steps of CBT.

As a tool for the ‘follow-up care’ at home, a wireless sensor monitoring system (see technical details of the SO-HIP tool) will be installed in the home of the participant on the first day after discharge from the nursing home for a period of 3 months. After being discharged, the participants also receive four home visits by an occupational therapist, which are then followed by four consultations by telephone, and in doing so, following the five steps mentioned above with the input of the sensor data, according to the same structure.

The contents of the different sessions are described in a manual for the occupational therapist.

#### Training and education of the trial occupational therapists

All occupational therapists of the two intervention groups in the nursing homes will receive information about the study, including a manual with the procedures and a two-day training session (first day before the start of OTc and the second day before the start of OTcsm) regarding how to make use of the CBT principles in coaching the participant and how to make use of the SO-HIP tool in instructing and coaching the patient (face to face and by telephone), following the five steps of CBT. Along with the coaching on the use of the sensor data, the occupational therapists will be instructed about the technical aspects of the SO-HIP tool and the use of the web-based application. Details of the training program can be found at www.sohipstudie.nl. The occupational therapists are all registered, have a bachelor’s degree and have experience in the rehabilitation of patients after hip fracture.

### Use of co-interventions

Patients are allowed to receive concurrent interventions during the study period (e.g., medications, dietician). Details of the concurrent intervention(s) will be registered.

### Outcome and measurements

Table 3 gives a detailed overview of outcome measures at each time point.

**Table 3:** Variables and outcome measures and time points of assessment in the SO-HIP study

Measures	Baseline NH1	T1 NH2	T3 H1	T6 H2
Primary outcome measure				
Daily functioning; self perceived performance in daily activities:				
o COPM	X	X	X	X
Secondary outcome measures				
Physical functioning;				
o Performance oriented mobility: POMA	X	X	X	X
o Functional mobility and balance: TUG	X	X	X	X
Independence in Activities of Daily Living (ADL) and Instrumental Activities of Daily Living (IADL;				
o Katz-15 index	X	X	X	X
Sense of safety;				
o VAS-SAFE	X	X	X	X
Fear of falling;				
o VAS-FOF	X	X	X	X
o FES-I	X	X	X	X
Health related quality of life;				
o EQ 5D	X	X	X	X
Additional measures				
Information gathered of determinants of functional decline (e.g., comorbidities) and a minimal data set (MDS) consisting of;				
o Demographic data,	X			
o Psychological and social functioning; subscale Rand 36	X	X	X	X
o Cognitive functioning; MMSE	X			X
o Healthcare utilization	X		X	X

Baseline, NH1 = within 1 week after admission nursing home; T1, NH2 = before discharge from nursing home; T3, H1 4 months (post-intervention) at home; T6, H2 = 6 months after the start rehabilitation. *COPM* Canadian Occupational Performance Measure, *POMA* Performance Oriented Mobility Assessment, *TUG* Timed Up and Go, *Katz 15 index* Modified Katz 15 index, *VAS-SAFE* Visual analogue scale for sense of safety, *VAS-FOF* Visual analogue scale for fear of falling, *FES-I* Falls Efficacy Scale International, *EQ5D* EuroQol health related quality of life, *MDS* Minimal Dataset, *MMSE* Mini Mental State Examination

### Medical and demographic variables

The self-reporting questionnaire that participants fill out at baseline and T4 contains determinants of functional decline (e.g. comorbidities) and the elements of a minimal data set (www.topics-mds.eu) consisting of demographic data (e.g. age, gender, marital status), physical functioning, self-perceived health status, psychological and social functioning, health-related quality of life and health care utilization.

### Primary outcome measure

The primary outcome measure is the perceived daily functioning 6 months after the start of rehabilitation compared to baseline functioning (the first week after admission). The primary outcome measure will be measured using the Canadian Occupational Performance Measure (COPM) [35]. The COPM is a client-centered, occupation-focused outcome measure for the detection of change in perceived occupational performance over time. It is a generic measure suitable for all clients with perceived problems in daily activities. It uses a semi-structured interview format and a structured scoring method. The COPM results in two main scores, Performance and Satisfaction, each out of total of 10. The patient prioritizes up to five problems s/he deems that are the most urgent or important and rates the problems on an ordinary 10-point scale regarding performance (1 = not able to do at all and 10 = able to do extremely well) and satisfaction (1 = not satisfied at all and 10 = extremely satisfied). The mean scores will be obtained by summing the ratings for performance and satisfaction and dividing them by the number of prioritized problems. Change in scores can be calculated after a reassessment interval to measure the change in the perception of occupational performance. For evaluation at a later time, the patient rates the performance regarding the prioritized problems outlined in the first interview. The COPM is a standardized instrument, with specific instructions and methods for administering and scoring. The reliability and validity of the COPM have been shown in many studies, and the COPM is widely used as an outcome measure for individuals and interventions [36–40]. A 1.3-point difference between pre- and post-measurement indicates a minimally clinically important difference [39, 40].

In this study, a trained research assistant will do the COPM interview and score the results.

### Secondary outcome measures

The secondary outcome measures are the level of physical activity and independence in activities of daily living, the level of sense of safety, fear of falling, self-rated health and the use of healthcare resources at 1, 4 and 6 months after start of the rehabilitation, compared to functioning at baseline at the beginning of rehabilitation in the nursing home.

*Physical functioning* will be measured based on the following:

1. *Performance oriented mobility* will be measured using the Tinetti Performance Oriented Mobility Assessment (POMA). The POMA is an easily administered, generic and widely used task-oriented test that measures the gait and balance abilities of older adults and their association with the risk of falling (high risk of falls (Tinetti score ≤18 points), moderate risk of falls (Tinetti score between 19 and 23 points), and low risk of falls (Tinetti score ≥24 points) [41]. It is clinically used to determine the mobility status of older adults or to evaluate changes over time. The POMA score ranges from 0 to 28, with a higher score indicating better balance and walking ability [41]. The inter-rater and test–retest reliability of the POMA is excellent, and the correlation with reference performance tests indicates the satisfactory construct validity of the POMA [42].
2. *Functional mobility and balance* will be measured by the Timed Up and Go (TUG). The amount of time to rise from a chair with arm rests, walk 3 meters, cross a line on the floor, turn, walk back, and sit down again will be measured [43]. The test will be performed twice, and the mean time will be used as the outcome [44]. The TUG range for people aged 80 to 99 years expressed as the mean has been estimated to be 11.3 ((95% confidence interval10.0-12.7) seconds [45] and 11 to 20 s in frail elderly and disabled patients [43]. The TUG is well validated and has been used in several studies on hip fracture patients to predict falls, to assess functional mobility and to assess the effects of home-based therapy and comprehensive geriatric care [3, 43, 46–48].
3. *Independence in Activities of Daily Living (ADL) and Instrumental Activities of Daily Living (IADL)* will be measured using the modified Katz-ADL 15 index score. This index is based on six basic ADLs and nine IADL items. Each item is scored 0 (independent) or 1 (dependent), with an overall score ranging from zero to 15; a higher score indicates a higher dependence in ADL and IADL [49, 50].

#### Sense of safety

The visual analogue scale for sense of safety (VAS-SAFE) will be used to measure sense of safety levels. The respondents answer the question “How safe do you feel at home?” The participants are instructed to select the number that best reflects their perceived sense of safety, with 1 representing feeling safe and 10 representing feeling extremely unsafe.

*Fear of falling* will be measured with the visual analogue scale for fear of falling (VAS-FOF) and the Falls Efficacy Scale International (FES-I).

1. The VAS-FOF is a simple and easy-to-use instrument that uses a numeric scale (1–10) to measure the perceived FOF. The participants are instructed to select the number that best reflects the intensity of FOF experienced, with 1 representing no FOF and 10 representing an extreme FOF [51].
2. The Falls Efficacy Scale-International (FES-I) is a short, easy-to-administer tool that measures the level of fear of falling during social and physical activities inside and outside the home, whether or not the person actually does the activity. The level of concern is measured on a four-point Likert scale (1 = not at all concerned to 4 = very concerned) [52].

The reliability and structural validity of the FES-I in patients after a hip fracture are good [53]. The Falls Efficacy Scale-International (FES-I) is commonly used to the measure fear of falling in community-dwelling older adults but can also be used to assess the fear of falling in patients after hip fracture [38].

#### Health-related quality of life

Self-reported health-related quality of life will be measured with the EQ 5D (EuroQol), comprising a visual analogue scale (VAS) and a health status instrument. EQ-5D is a validated, generalized and standardized instrument for use as a measure of health outcome. The EQ 5d compromises the following 5 dimensions: mobility, self-care, activities, pain/ discomfort and anxiety/depression, and one question about cognition. Each dimension has three levels: no problems, some problems or extreme problems [54]. A respondent’s EQ-VAS indicates self-rated health on a scale in which the endpoints are labeled ‘best imaginable health state’ (100) and ‘worst imaginable health state’ (0).

It was found that the EQ-5D could be used to measure outcomes for patients recovering from hip fracture, including those with cognitive impairment [55].

### Process evaluation

In addition to the primary and secondary outcomes, additional qualitative data will be collected, which will give insight into the feasibility of the SO-HIP tool at the level of both the older participants after hip fracture and the professionals using this intervention. Participants’ experiences and opinions with the standard care, OTc and OTcsm will be evaluated in a qualitative study, which will be done alongside the feasibility study of the SO-HIP study. From the professionals we will collect data using standardized evaluation forms. For each participant, each therapist will record the content of their intervention, the number of sessions, time spent and their views of effectiveness of the intervention. At the end of the study we will conduct a focus group with all professionals involved in the study exploring their experiences and opinions regarding the use of coaching and the use of coaching combined with sensor monitoring.

### Sample size calculation

Stepped wedge designs with more than two interventions have, to our knowledge, never been reported. The methodology for sample size and power calculations are still being developed. Dr. Steven Teerenstra, PhD (Biostatistics, Radboud University Medical Center) performed a simulation-based power calculation based on the primary outcome – the COPM performance outcome. Specifically, with 8 patients per cluster (nursing home) per step (six steps of 2 months duration each), an assumed treatment effect 1 (occupational therapy without sensor monitoring (OTc) versus usual care (control, C) of 1.5*SD) and an assumed treatment effect 2 (occupational therapy with sensor monitoring (OTcsm) versus OTc of 0.75*SD), and an intracluster correlation coefficient of 0.05, we will collect observations on 288 patients and achieve a power of 100% for treatment effect 1 and a power of 85% for treatment effect 2. We expressed the treatment effect sizes relative to the standard deviations (SD) because similar data are currently lacking.

### Data entry and quality control

We will collect the data using standardized forms and measurements. A trained research assistant will collect data at baseline (T0), before discharge from the nursing home (T1), 4 months (post-intervention) (T3) and at 6 months (follow-up) (T4). All data will be entered into a database (Castor, http://castoredc.com), according to Academic Medical Centre Good Clinical Practice Guidelines with an identification code for each patient.

The sensor monitoring data of the patient will be kept under the identification code and stored in a secured database.

According to the good clinical practice guidelines, data will be stored for 15 years and archived according to the regulations of the Netherlands Federation of University Medical Centers (NFU) (http://www.nfu.nl).

### Statistical analysis

An adapted CONSORT flow diagram will detail the flow of clusters and patients through the trial (see Fig. 1). Baseline comparability at the level of clusters (immediately after randomization) and patients (at recruitment) will be assessed. Descriptive data will be used to assess any time trends of patient characteristics at recruitment since patient selection bias is a threat in cluster trials that cannot be blinded for allocation.

The treatment effects (OTc vs control, OTcsm vs OTc, and OTcsm vs control) on the various outcomes will be estimated with mixed linear models using dummy variables for the two treatments, random intercepts for the clusters, and time as a fixed effect. For each outcome, the baseline values of that outcome will be used as a covariable [56]. The trial will have limited power to explore treatment by time or treatment by cluster interactions. If feasible, we will explore these. Two sided 95% confidence intervals will be calculated.

An intention-to-treat analysis will be the primary analysis. Per-protocol analyses based on degree of compliance with the study protocol will be used in an exploratory fashion.

A descriptive qualitative and quantitative analysis will be conducted on the data from the evaluation forms of the participants and the assessors and the data from the therapists of a given intervention. We will analyze the qualitative data based on the constant comparative method [57].

## Discussion

The present three-arm stepped wedge randomized trial combines CBT principles that have been successful in the treatment of fear of falling and the multidisciplinary rehabilitation of older adults with hip fracture with the incorporation of sensor monitoring in the intervention as a coaching tool (monitoring and feedback tool) to improve daily functioning, physical activities, sense of safety and reduce the fear of falling at home. To our knowledge, this is a first trial evaluating the effectiveness of these interventions in older individuals after hip fracture.

Stepped wedge designs with more than two interventions have, as far as we know, never been used. Because we make use of restricted randomization we will reduce the between-cluster variation and improve balance, which is advisable when there are few clusters [58].

The use of a stepped wedge design provides us some methodological and practical advantages. First, the intervention effect can be estimated using between and within cluster comparisons and the professionals are their own controls in the interventions [59]. Second, each participating nursing home will have implemented both interventions at the end of the study while in a traditional cluster randomized trial some clusters will have received only a control intervention. This increased nursing homes’ willingness to participate. Third, in order to provide training in each cluster before the start of the interventions, the staggered start of the interventions makes a better time allowance. The same accounts for the technical support of the tool if needed. Last, because of the crossover from control to OTc and OTcsm and each participant receives only one condition, we may assume that there are no carryover effects [58].

For older adults, the ability to remain mobile is an essential aspect of quality of life and is crucial for the preservation of independence [15]. An important aspect of the intervention using sensor monitoring is to apply CBT principles. Sensor monitoring embedded in the OT intervention with CBT coaching is expected to have an impact directly at the level of the patient’s ability to perform activities in his or her own context. A characteristic of the use of sensor monitoring in an OT intervention is that goals related to daily activities are formulated that are relevant and important to the person and are based on the objective measurement of daily functioning by sensors. The coaching by the occupational therapist will target these particular issues. Our hypothesis is that the person’s self-perceived performance in daily activities, measured using the COPM, will alter as a result of the intervention.

This study will provide new knowledge regarding the combined intervention of CBT coaching by occupational therapists and CBT coaching by occupational therapists using sensor monitoring, enabling older individuals to perform everyday activities and to remain living independently after hip fracture.

## Abbreviations

ADL: Activities of daily living
CBT: Cognitive behavioral treatment
COPM: Canadian Occupational Performance Measure
EQ5D: Euroquol, instrument for assessing health related quality of life
FES-I: Falls Efficacy Scale-International
IADL: Instrumental Activities of Daily Living
Katz-15: Katz Index of Independence in Activities of Daily Living
MDS: Minimal dataset
MMSE: Mini Mental State Examination
OT: Occupational therapy
OTc: Occupational Therapy with Coaching
OTcsm: Occupational therapy with coaching and sensor monitoring
PAM: Physical Activity Monitor
POMA: Performance Oriented Mobility Assessment
PT: Physical therapy
SO-HIP: Sensor monitoring embedded in Occupational therapy rehabilitation for patients after Hip-fracture
SO-HIP tool: Sensor monitoring system used for hip rehabilitation
VAS-SAFE: Visual Analogue Scale for Fear of Falling
WMO: Medical research involving human subjects act
